# Supplementary material for: The genome sequence of an ichneumonid wasp, Diplazon laetatorius (Fabricius, 1781)
Source: Wellcome Open Res. 2024 May 15;9:270. [Version 1] doi: 10.12688/wellcomeopenres.21626.1 (PMC12515358; doi:10.12688/wellcomeopenres.21626.1)
Supplement: Peer review history

# Reviewer report: Reviewer response for version 1

**DOI:** 10.21956/wellcomeopenres.23898.r127147

**Published:** 2025-10-11

**Authors:** Julien Varaldi, Sara Oukkal

**Affiliations:** 1 Universite Claude Bernard Lyon 1, Villeurbanne, France; 2 UMR CNRS 5558 - LBBE, Universite Claude Bernard Lyon 1, Villeurbanne, Auvergne-Rhône-Alpes, France

This manuscript presents a chromosome-level genome assembly for the parasitic wasp *Diplazon laetatorius*. The genome is of high quality and the first available for this genus. We have a few minor comments that we hope will help improve the clarity of the manuscript:

**Introduction :**

 The term “stout insect” is unclear to us, could you clarify what is meant by this?

 There is a typo in the sentence “has been able to able establish”.

**Results :**

 In Figure 3, it is mentionned that sequences are coloured by phylum. However, only Arthropoda (or no hits) appear to be depicted. Does it mean that there is no contigs assigned to any other phyla?

 Figure 4 is not very useful and could possibly be removed.

 In the Hi-C plot, we noticed a possible issue with chromosome-level scaffold 11. It seems that the second and third blocks need to be swapped. Possibly the same artefact occurs on chromosome 2.

 It is also unclear whether chromosome 3 is a single chromosome or should be split in two.

 No information on the fold coverage of Hi-C reads is provided. Please add this information.

**Methods :**

 Please clarify what is meant by “three domain-level BUSCO lineage”. 

 Could you also clarify the meaning of this sentence “The genome is also split into chunks according to the density of the BUSCO genes from the closest taxonomically lineage, and each chunk is aligned to the Uniprot Reference Proteomes database with DIAMOND blastx” 

 The RNAseq data are mentioned as already available for future functional annotation, but there is no indication of which data this refers to (accession number?).

 It would be very helpful to provide a GitHub repository with the full analysis pipeline. Several external pipelines are cited, but it is difficult to identify exactly which scripts were used in this study and in what order.

 We hope these suggestions are useful.

Are sufficient details of methods and materials provided to allow replication by others?

Yes

Is the rationale for creating the dataset(s) clearly described?

Yes

Are the datasets clearly presented in a useable and accessible format?

Yes

Are the protocols appropriate and is the work technically sound?

Yes

Reviewer Expertise:

insect evolutionary genomics

We confirm that we have read this submission and believe that we have an appropriate level of expertise to confirm that it is of an acceptable scientific standard.

# Reviewer report: Reviewer response for version 1

**DOI:** 10.21956/wellcomeopenres.23898.r102276

**Published:** 2024-10-02

**Authors:** Zachary Lahey

**Affiliations:** 1 USDA-ARS, Washington, D.C., USA

The purpose of this article is to report the genome assembly of *Diplazon laetatorius*, an ichneumonid wasp. The manuscript is well-written and easy to follow. Most of the manuscript is methodological, allowing one to reproduce the analyses reported.

 I have three comments that need to be addressed:

 1. In the Genome Sequence Report section, a comment is made that contigs from the second sequenced haplotype have been deposited, but it is not mentioned where.

 2. Under Isolate information in Table 1, the isolate identifier is repeated twice, presumably because two specimens were sequenced. The isolate identifiers, however, are identical, so the isolate identifier should only be mentioned once.

 3. Figures 3 and 4 don't add anything to the quality of the manuscript, in my opinion, and would be better off removed or consolidated into a single figure that could be uploaded as a supplement.

Are sufficient details of methods and materials provided to allow replication by others?

Yes

Is the rationale for creating the dataset(s) clearly described?

Yes

Are the datasets clearly presented in a useable and accessible format?

Yes

Are the protocols appropriate and is the work technically sound?

Yes

Reviewer Expertise:

genomics; phylogenetics; systematics

I confirm that I have read this submission and believe that I have an appropriate level of expertise to confirm that it is of an acceptable scientific standard.
